# Longitudinal investigation of undergraduates’ radiation anxiety, interest, and career intention in interventional radiology

**DOI:** 10.1007/s00330-024-10848-8

**Published:** 2024-06-21

**Authors:** Yanyan Cao, Li Yu, Fu Xiong, Jing Wang, Xuefeng Kan, Chuansheng Zheng

**Affiliations:** 1 Department of Radiology, Union Hospital, Tongji Medical College, Huazhong University of Science and Technology, Wuhan, Hubei 430022 China; 2 Hubei Province Key Laboratory of Molecular Imaging, Wuhan, Hubei 430022 China; 3 Department of Emergency Medicine, Union Hospital, Tongji Medical College, Huazhong University of Science and Technology, Wuhan, Hubei 430022 China

**Keywords:** Interventional radiology, Clinical exposure, Radiation anxiety, Professional interest, Career intention

## Abstract

**Purpose:**

To evaluate the effect of the school curriculum and on-site observation of interventional radiology (IR) operations in clinics on undergraduates’ radiation anxiety, interest, and career intention.

**Methods:**

Between the academic years 2021 and 2023, all of the fourth-year undergraduates were surveyed by questionnaires, which covered their pre-curriculum, post-curriculum in-school, and post-on-site view of IR surgeries in clinic. The survey included categories of gender, fear of X-ray and IR operation, interest in IR surgery, and career-pursuing intention.

**Results:**

A total of 333 (91.0%) respondents (111 students for three times) were included in analyses. The fear of X-ray and radiation exposure during IR procedures was reduced after taking school courses (*p* < 0.001), and it was further decreased after on-site viewing (*p* < 0.001). The association values among the three groups were 33.8% and 41.9%, respectively. The interest in IR was improved both after applying for the curriculum and after clinical exposure to IR surgery (*p* < 0.001). In addition, 4 (3.6%) and 12 (10.8%) students showed a sense of achievement after taking courses and on-site viewing, respectively. The association value was 49.4%. Regarding career intention, it was both significantly increased after taking courses and on-site observation (*p* < 0.001). Besides, 8 (7.2%), 17 (15.3%), and 36 (32.4%) students in the three groups considered IR as the preferred career choice, respectively.

**Conclusions:**

Applying for IR curriculum could reduce undergraduates’ radiation anxiety, and activate their professional interest and career pursuing intention. Clinical exposure to IR surgeries further boosted this effect.

**Clinical relevance statement:**

Educational interventions of curriculum and on-site view of IR surgery improve the undergraduates’ interest in IR and stimulate their career intention, which is crucial for the advancement of IR.

**Key Points:**

- *Increasing interest in interventional radiology (IR) as a career is urgent, given rising demand of services*.
- *Education and on-site viewing of IR surgery reduced radiation anxiety and increased interest in IR*.
- *Early exposure to IR is effective at encouraging undergraduates to consider IR as their career*.

## Background

Interventional radiology (IR) delivers minimally invasive treatments to a wide range of diseases to improve the quality of life and saving of patients’ lives, such as gastrointestinal and trauma-related bleeding, cancer therapy, repair of aortic aneurysms, obstructive jaundice, etc. In recent decades, IR has transformed the way of managing patients, becoming a fundamental medical subspecialty. It is rapidly expanding worldwide [1–3].

However, the recruitment of doctors into IR has lagged behind the demand for IR clinical development [4]. As future referring physicians or potential interventional radiologists, medical students are the core for the future development of IR. Therefore, adequate dissemination of IR among students and the arousal of their interest would promote their career selection intentions. However, previous surveys revealed poor knowledge of and limited exposure to IR among medical undergraduates [5–7].

Unlike in Western countries, Chinese undergraduates received a formal IR curriculum. However, the impact of medical school on their perceptions and career willingness warrants further investigation. Additionally, research has indicated that curriculum or symposia can boost students’ interest in IR to some extent, but it remains to be improved [4, 8, 9].

Considering these limitations, the purpose of this study was to compare undergraduates’ perceptions and career-pursuing intentions pre- and post-curriculum in China. Moreover, an on-site view of IR operations was further provided for these undergraduates, and a re-evaluation was conducted.

## Materials and methods

Research board approval was waived at our institution because the survey was anonymous and voluntary. A total of 366 questionnaires (122 questionnaires distributed each time, for a total of 3 distributions) were administered using an online software in WeChat to all fourth-year students enrolled in the Imaging and Nuclear Medicine major at a Chinese medical school between the academic years of 2021 and 2023. The participants included three batches of undergraduates. The same questionnaires were given to students three times: pre-curriculum, post-curriculum while pre-on-site view of IR operation, and post-on-site view.

An on-site view of IR operations was conducted by introducing these undergraduates to the IR operating room and observing entire procedures of different IR surgeries outside the room 1 week to 1 month after applying for courses. The curricular contents delivered in the classroom were included in the textbook. After arriving at the operating room, the teacher/surgeon provided students with an overview of surgical procedures, combining practical experience with theoretical knowledge from the curriculum. The surgical categories included transarterial chemoembolization, thermal ablation, peripheral vascular angioplasty, and embolization therapy for arterial bleeding, etc.

The questionnaire consisted of five questions with checkbox answers (electronic supplementary material). The survey included categories of gender, fear of x-rays, fear of IR operation, interest in IR surgery, and career-pursuing intention. Fear of x-rays was categorized into four levels: fearless, mild, moderate, and severe. Similarly, fear levels related to IR operations were also divided into these same categories. Interest in IR surgery included blind to IR, not interested in it, interest in it, and having a sense of achievement. Career pursuing intention was classified as no intention, would consider it as an alternative, and would select it as a preferred career path. Students were informed that the survey was completely anonymous, voluntary, and unrelated to any current or future assessments or career choices. The questionnaire was excluded from this study if the student refused to participate, the questionnaire results were incomplete, or the handwriting could not be accurately identified.

### Statistical analysis

All the data were categorical variables and expressed as percentages. G*Power software (GPower 3.1) was utilized for power calculation (1-β err prob) based on post hoc testing (sample size = 111, two-tailed testing, α = 0.05, effect size = 0.5). The resulting power value was 0.804, indicating that this sample size was appropriate. Other analyses were carried out using SPSS version 25.0 software (IBM, Armonk, NY, USA). If more than 20% of cells had theoretical frequencies (desired frequency) T < 5 or if at least one T < 1, the differences between groups were analyzed using Fisher’s exact test; otherwise, Pearson’s chi-square test was used. A two-tailed *p-*value of less than 0.05 considered statistically significant.

## Results

**Fig. 1:**
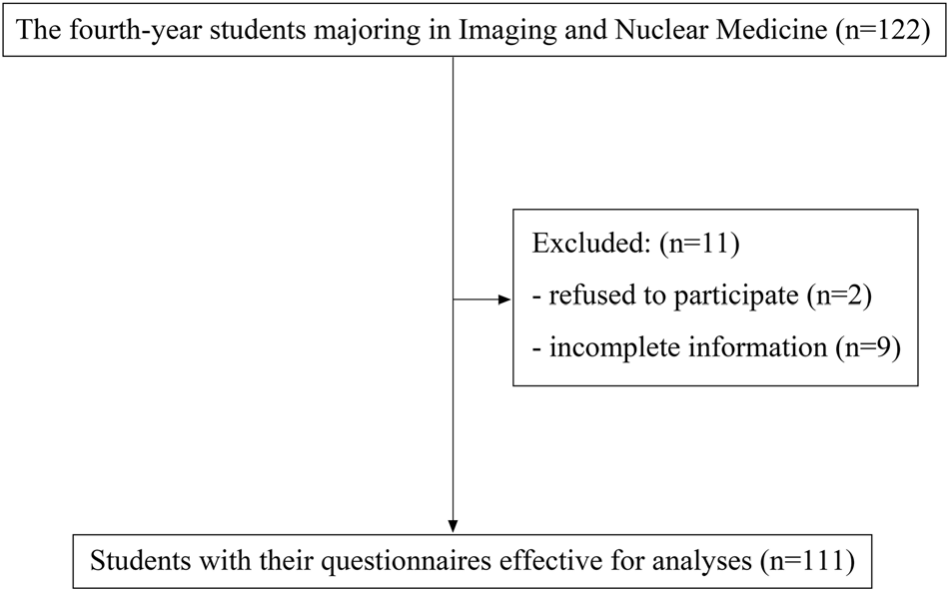
The flowchart of participants recruitment. A total of 111 students with their questionnaires eligible for analyses

A total of 333 (91.0%) questionnaires (111 for three times) were effective and used for analyses (Fig. 1). The remaining 11 (9.0%) respondents were excluded for refusing to participate or incomplete information. There were 66 (59.5%) males and 45 females (40.5%) among the effective respondents.

**Fig. 2:**
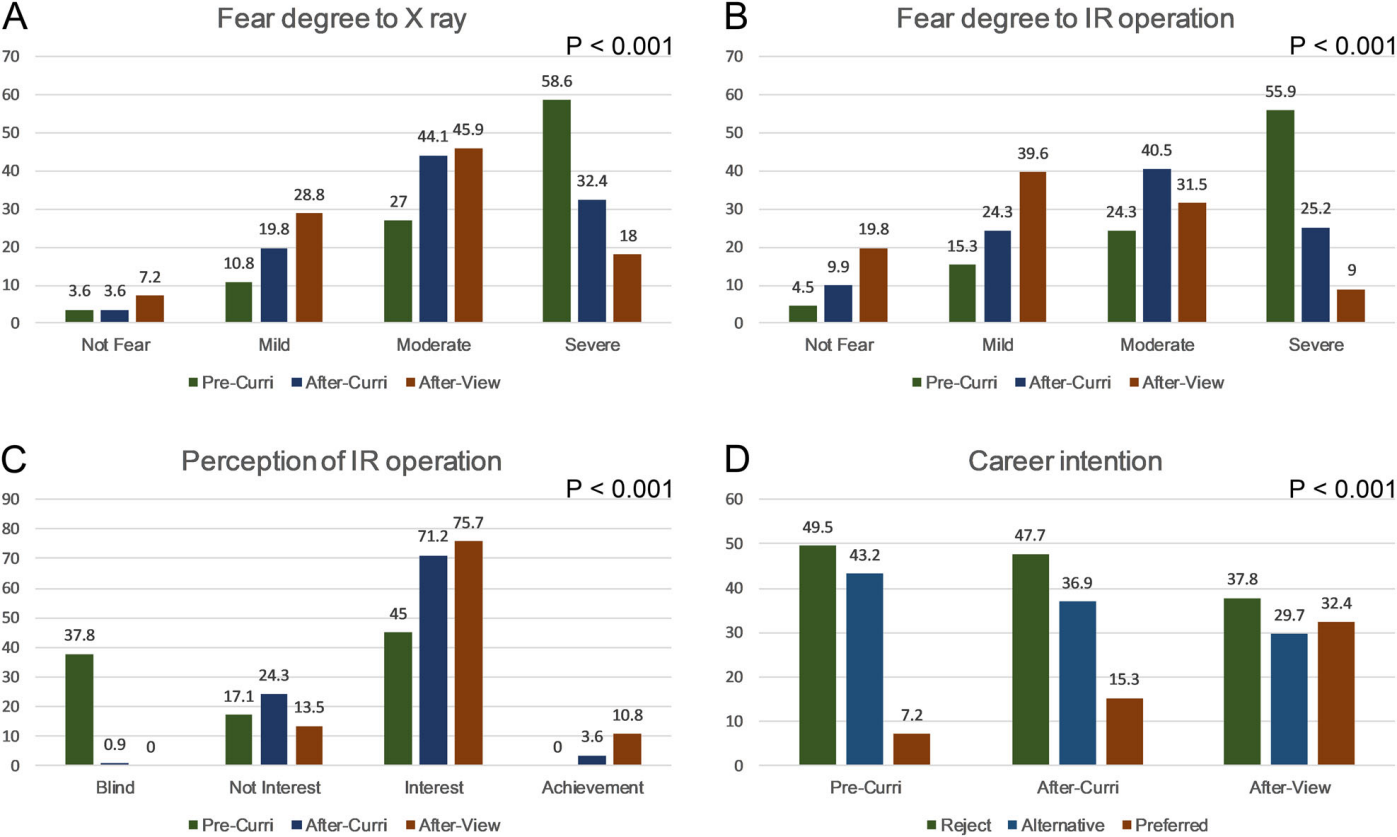
Changes in undergraduates’ radiation anxiety, perception of IR operation, and career pursuing intention. **A** The fear degree of X-ray pre- and post-applying for curriculum, and after on-site viewing of IR operations. The difference was significant (*p* < 0.001). **B** The fear degree of radiation exposure during IR surgery procedures (*p* < 0.001). **C** The perception of IR before and after applying for curriculum, and after on-site viewing of IR operations (*p* < 0.001). **D** The change of career pursuing intention pre- and post-applying for curriculum, and after on-site viewing of IR operations (*p* < 0.001). IR, interventional radiology

**Fig. 3:**
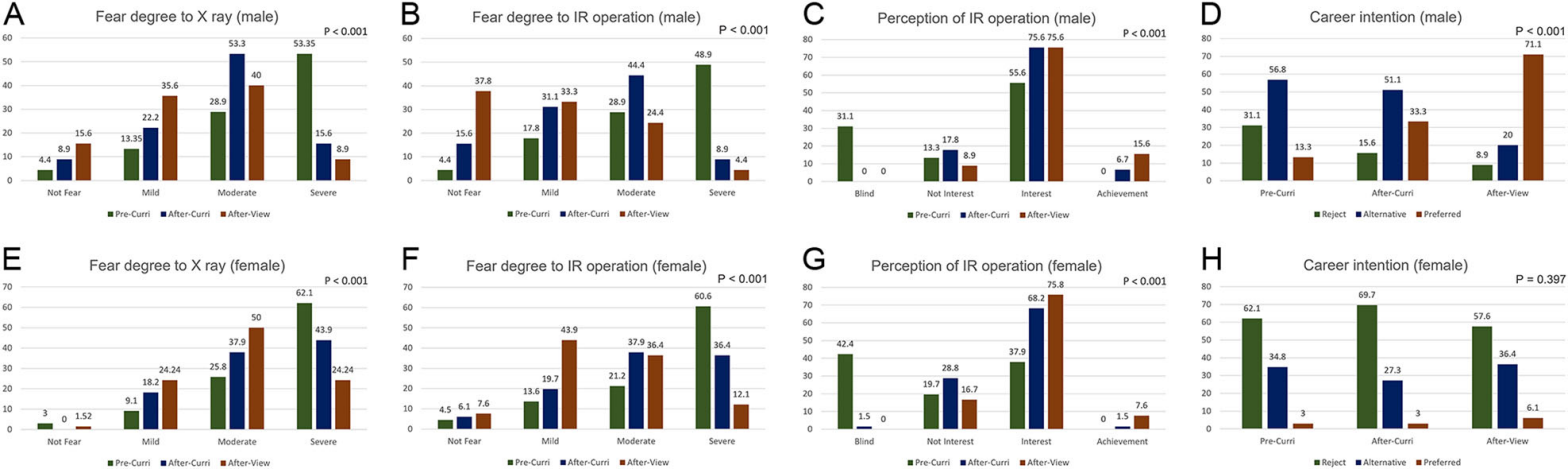
Changes in radiation anxiety, perception of interventional radiology (IR) operations, and career pursuit intentions among male and female undergraduates. **A**–**D** Improvement in the attitudes of male undergraduates towards radiation anxiety, IR profession, and career pursuit intentions. **E**–**H** Changes in the attitudes of female undergraduates towards radiation anxiety, IR profession, and career pursuit intentions. IR, interventional radiology

As illustrated in Fig. 2, the majority of students were afraid of X-rays before taking the course. The number of students with mild, moderate, and severe fear was 12 (10.8%), 30 (27.0%), and 65 (58.6%), respectively. After the curriculum, the degree of fear decreased. After on-site viewing of IR operations, it was further decreased to 32 (28.8%), 51 (45.9%), and 20 (18.0%), respectively. The difference among the three groups was statistically significant (*p* < 0.001). In the gender-specific subgroup analyses, the results (Fig. 3) showed that the difference among males was also statistically significant (*p* < 0.001), and so was it among the females’ subgroup (*p* < 0.001).

Regarding radiation exposure during IR surgery procedures, 5 (4.5%), 11 (9.9%), and 22 (19.8%) students expressed fearlessness in the pre-curriculum, post-curriculum, and post-on-site view groups, respectively. The mild degree increased, and the severe degree decreased after taking courses in school and observing IR operations on-site. These changes presented statistically significant differences among these groups (*p* < 0.001). In gender-specific subgroup analyses (Fig. 3), statistically significant differences were observed among males (*p* < 0.001), as well as among females (*p* < 0.001).

There were 42 (37.8%) students blind to IR before applying for the curriculum, while almost all of them understood what it was after taking school courses. There were 19 (17.1%), 27 (24.3%), and 15 (13.5%) students who had no interest in IR pre-curriculum in the three groups, respectively. The number of undergraduates who expressed interest post-curriculum in the three groups was 50 (45.0%), 79 (71.2%), and 84 (75.7%), respectively. Furthermore, 4 (3.6%) and 12 (10.8%) students expressed a sense of achievement after applying for courses and observing the IR surgeries on-site, respectively. The change of interest was significantly different among the three groups (*p* < 0.001). In the gender-specific subgroup analyses, there were 25 (55.6%), 34 (75.6%), and 34 (75.6%) male undergraduates expressed interest in the three groups, respectively. The number of male students expressing a sense of achievement also increased in these groups from 0 (0%), to 3 (6.7%), and then to 7 (15.6%) respectively (*p* < 0.001). The number of female students who expressed interest in IR was also increased from 13 (19.7%), 19 (28.8%), and 11 (16.7%) to 25 (37.9%), 45 (68.2%), 50 (75.8%), respectively. Additionally, five females showed a sense of achievement after on-site viewing of IR operations. The difference among the female subgroup was statistically significant (*p* < 0.001).

Regarding career intention, 55 (49.5%) undergraduates did not want to pursue a career in IR prior to the curriculum. Fifty-five (47.7%) had the same attitude after taking courses in school, and the number dropped to 42 (37.8%) after watching operations in the clinic. There were 48 (43.2%), 41 (36.9%), and 33 (29.7%) students would consider IR as an alternative career in the three groups, respectively. In addition, 8 (7.2%), 17 (15.3%), and 36 (32.4%) students chose IR as their preferred career path, respectively. The difference was statistically significant among the three groups (*p* < 0.001). However, after on-site viewing, the majority of undergraduates who chose IR as their preferred career path were males (*n* = 32, 88.9%), while only four females did so (11.1%). Additionally, the difference among the three male subgroups was statistically significant (*p* < 0.001), but not among female subgroups (*p* = 0.397).

**Fig. 4:**
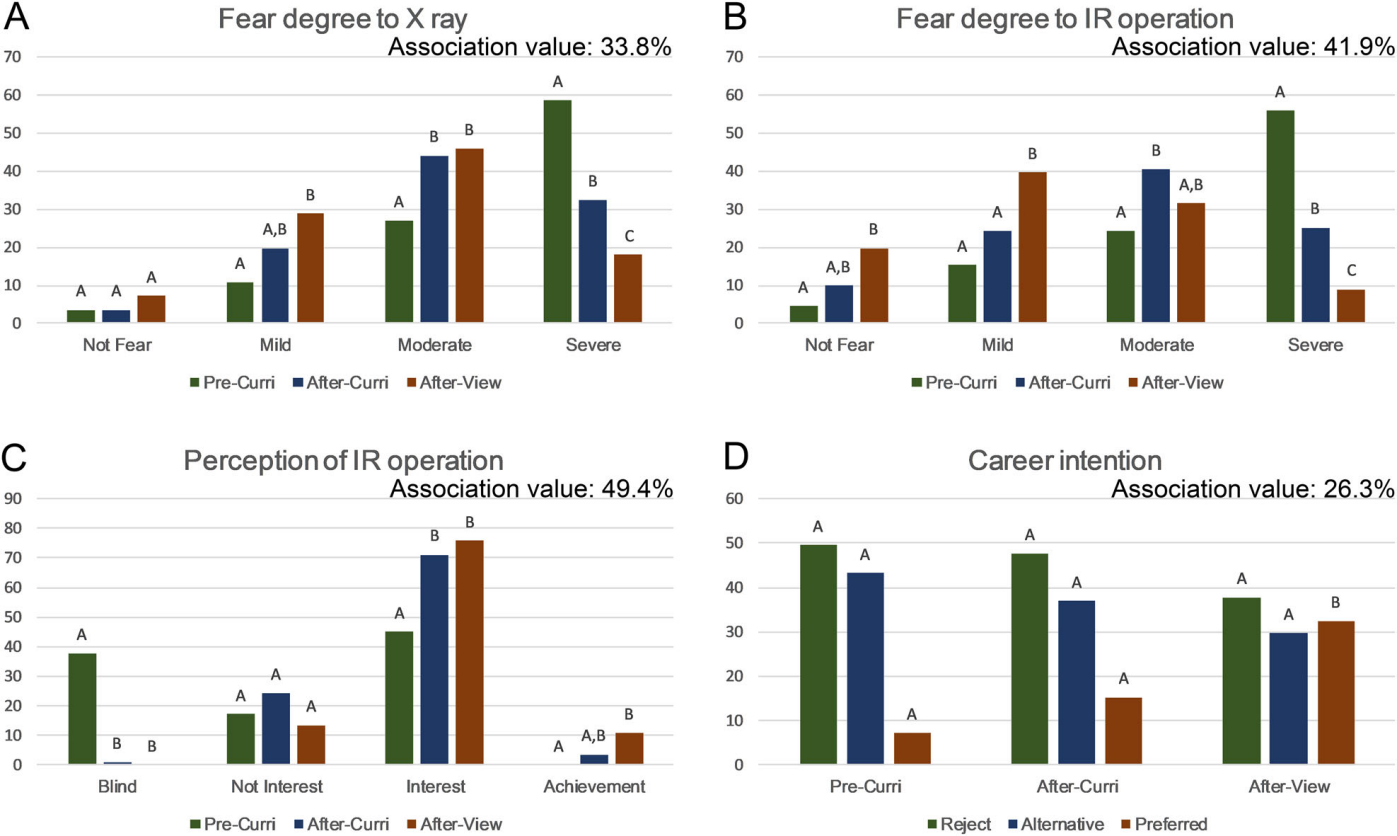
Correlation between the changes in undergraduates’ attitude and the intervention of curriculum or on-site view of IR surgeries. **A** Group comparation of the undergraduates’ anxiety to X-ray before and after applying for curriculum, and after on-site viewing of IR operations. The association value was 33.8%. **B** Group comparation of the undergraduates’ fear to IR operation. The association value was 41.9%. **C** Group comparation of the undergraduates’ perception of IR operation. The association value was 49.4%. **D** Group comparation of the career pursuing intention. The association value was 26.3%. Note: The distinct capital on the bar graph (different group) indicated the difference was significant. For example, the difference in the sense of achievement was significant in the post-on-site view group compared with the pre-curriculum group (**A** vs **B**). However, it was not significant compared the pre-curriculum group to the post-curriculum group (**A** vs **A**, **B**). In addition, the association value refers to the strength of the relationship between educational interventions and changes in attitudes. A larger value indicates a closer relevance between changes in undergraduates’ attitudes and educational interventions in curriculum and on-site view

To further investigate whether these significant changes were related to the course taken in school and the on-site clinical exposure, the associations between them were evaluated. As shown in Fig. 4, students with a mild fear of X-ray increased significantly after on-site viewing compared to that of before taking courses, while it was nonsignificant only after curriculum. However, after applying for curriculum in school, the number of students with moderate fear increased significantly, while the number with severe fear was significantly reduced. In addition, the number of severe fear students was further significantly reduced after watching IR operations. The association value among these groups was 33.8% (The larger values indicate closer relevance between changes in undergraduates’ attitudes and educational interventions through curriculum and on-site view).

The number of undergraduates who did not fear IR operation after on-site viewing was significantly improved compared to the pre-curriculum group, while it was not statistically significant after taking school courses. Following the IR surgeries, a statistically significant increase in the number of students with mild fear was also observed. The number of moderate fear students significantly increased after applying for courses, while it was decreased for severe fear students. Additionally, the number of students with severe fear further decreased after on-site viewing. The association value among these groups was 41.9%.

After completing the curriculum, the students sharply enhanced their knowledge of IR surgery, as was their interest. In particular, the sense of achievement was significantly improved after on-site viewing of IR operations. The association value between these groups was 49.4%.

As for career intention, the number of undergraduates who chose IR as their preferred career was significantly increased after on-site viewing compared to before and after taking courses. The students who did not want to pursue a career or who considered IR as an alternative career did not change significantly. The association value between these groups was 26.3%.

## Discussion

Previous studies indicated that there is the need for additional efforts and innovative methods to reach out to students who are potentially interested and motivated [8]. This study longitudinally compared the undergraduates’ radiation anxiety, interest, and career intention to IR by investigating the changes before and after curriculum, as well as after on-site viewing of IR operations. Such a method is uncommon in this research field as far as we know, because the majority of the studies are cross-sectional. Moreover, the sense of achievement and preferred career intention for IR were categorized in our study, with the aim of identifying undergraduates who are most likely to pursue IR as a career.

With rising demand for IR services, the need for expansion of the workforce globally is inevitable [10]. On the other hand, IRs have lost “turf wars” to cardiologists [11]. Therefore, it is urgent to arouse undergraduates’ intention to pursue IR as a career. Previous studies indicated that students’ interest was increased after an IR rotation [12, 13]. However, the reality in China differed from that of most other countries. Undergraduates in our country who major in medical radiology apply for formal IR courses in school, and they choose their postgraduate specialty once they have completed their bachelor’s degree. Furthermore, the majority of their careers were in line with their postgraduate specialties. Therefore, it is critical to cultivate an interest or intention in IR during the undergraduate years. A previous study suggested that rational early experiences could strengthen cognitive abilities, broaden affective understanding, contextualize knowledge, and integrate medical education [14]. The survey from the European Society of Radiology and Cardiovascular and Interventional Radiological Society of Europe also indicated that IR needs to be recognized early as a possible career option to attract students [15]. Our results demonstrated that not only was the school curriculum effective, but the clinical on-site view of IR surgery could further promote their choice. Adult learning theories provide a potential theoretical basis for our conclusion that clinical environments are ideal for learning [16], combining theoretical concepts with practical elements where feedback is immediate. In line with this, the teaching effect was further improved in our study.

Although the learning process is complex, experiential learning can help enhance clinical performance and reduce learner anxieties [17–20]. Studies have also indicated that a lack of general knowledge and interest in radiology is the primary reason for not considering a career in IR [4, 21], radiation exposure during IR procedures could also discourage students in China from choosing IR as a career [11]. However, exposure to IR would increase the respondents’ intention [10, 12]. Our results were somewhat consistent with this conclusion, revealing that before applying for the curriculum, more than 58% and 55% of participants were extremely afraid of X-ray and radiation during the IR procedure, respectively. However, this phenomenon was obviously improved after taking school courses, both in male and female subgroups. Moreover, the fear was further alleviated after on-site viewing of IR surgeries, which had not been reported as far as we know. These results indicated that radiation anxiety was primarily attributed to a lack of knowledge, which could be effectively mitigated by having a close interaction with the IR operation.

In accordance with previous research, the interest in IR could be improved after applying for IR courses, despite of that interest varies by country [5, 22, 23]. Our study also presented that after applying for the curriculum, the number of students interested in IR increased from 45.0% to 71.2%, which was consistent with another Chinese survey, which found that approximately 68.4% of students were interested in IR. Moreover, the number increased significantly to 75.7% after on-site observation in our study. In addition, our survey further investigated the students’ sense of achievement, and the result showed that 4 (3.6%) of them expressed such a sense after completing courses. Surprisingly, this number tripled (10.8%) after on-site observation. These improvements were observed in both male and female subgroups. Besides, the association value of IR surgery anxiety and interest in IR operation reached 41.9% and 49.4%, respectively, indicating a close relevance between changes in undergraduates’ attitudes and educational interventions. These results demonstrated the positive impact of the curriculum and on-site experience, particularly of the clinical IR exposure, for both male and female students.

Regarding the different levels of interest, our survey included the options of alternative intention and preferred intention. Although the alternative career intention was reduced, the preferred choice was doubled after taking school courses according to our results. Additionally, the number was increased to 4 times after on-site viewing of IR operations. These results demonstrated again that not only the curriculum, but also the clinical exposure of IR was effective in increasing these undergraduates’ career intentions. On the other hand, the findings indicated that the on-site observation of IR operations activated these undergraduates’ potential interest and motivation to pursue careers. However, the majority of candidates for IR are male students who were primarily influenced to choose IR as their preferred career after on-site exposure, while few females did so. This suggests that despite a reduction in radiation anxiety and an increase in interest in the profession following educational interventions, women remain hesitant to pursue a career in IR. It is important to note that the underrepresentation of women in IR workforce is a global issue [15, 24, 25]. In addition to habitus, teaching methods also play a role in shaping career trajectories [24, 25], and increasing and optimizing mentoring opportunities may be the first step in maximizing potential female recruits [24]. Furthermore, targeted educational and policy initiatives aimed at female groups are essential [26].

There were several limitations in the study. First, this is a single-center and small sample study, which limits the generalizability of the findings to some extent. Second, the actual careers of these undergraduates have not been investigated, since the majority of them are still completing their degree, which will be studied in the future. Third, it is important to acknowledge the limitations of questionnaire-based studies including potential response bias and social desirability bias.

## Conclusions

Applying for an IR curriculum could reduce undergraduates’ radiation anxiety, and increase their interest in this profession and career pursuing intention. Moreover, on-site viewing of IR surgery could further increase their interest and career-pursuing intention, particularly for those who are potentially interested. This study offers a rational and effective approach for recruiting undergraduates considering IR as a potential career, which is critical for the development of IR.

## Supplementary information

ELECTRONIC SUPPLEMENTARY MATERIAL

## Abbreviation

IR: Interventional radiology
