# Supplementary material for: Longitudinal investigation of undergraduates’ radiation anxiety, interest, and career intention in interventional radiology
Source: Eur Radiol. 2024 Jun 21;34(12):7797–803. doi: 10.1007/s00330-024-10848-8 (PMC11557623; doi:10.1007/s00330-024-10848-8)
Supplement: Supplementary file 1 — ELECTRONIC SUPPLEMENTARY MATERIAL [file 330_2024_10848_MOESM1_ESM.pdf]

Longitudinal investigation of undergraduates' radiation anxiety, interest, and career intention in interventional radiology
ELECTRONIC SUPPLEMENTARY MATERIAL

The questionnaire was translated as follows:

1. Gender
- ○ Male
- ○ Female

2. Fear of X-ray
- ○ Fearless
- ○ Mild
- ○ Moderate
- ○ Severe

3. Fear of interventional radiology operation
- ○ Fearless
- ○ Mild
- ○ Moderate
- ○ Severe

4. Interest in interventional radiology surgery
- ○ Blind to it
- ○ Not interest in it
- ○ Interest in it
- ○ Have a sense of achievement

5. Career pursuing intention
- ○ No intention
- ○ Would consider it as an alternative choice
- ○ Would choose it as a preferred career path
